# Human Trafficking in the Emergency Department: Improving Our Response to a Vulnerable Population

**DOI:** 10.5811/westjem.2020.1.41690

**Published:** 2020-04-16

**Authors:** Jennifer Tiller, Stacy Reynolds

**Affiliations:** Levine Children’s Hospital and Carolinas Medical Center, Department of Emergency Medicine, Division of Pediatric Emergency Medicine, Charlotte, North Carolina

## Abstract

Human trafficking is a human rights violation affecting millions worldwide. Victims may go unrecognized during their emergency department (ED) visit, and may lose the opportunity to address their complex needs. Using a published toolkit based on existing guidelines and recommendations from experts, and models from other centers, we describe the implementation of an ED response protocol. In following the recommendations of the toolkit, we began with attempts to fully understand the local human trafficking problem and then networked with those working in anti-trafficking efforts. Collaboration with other specialties is highlighted as a key part of this process. Building upon the knowledge gained from these steps, we were able to develop a concise protocol to guide members of our department in more effectively caring for known or suspected victims of human trafficking. The first section of the protocol addresses ways in which providers can identify at-risk patients through both screening questions and general observations. Interviewing techniques are outlined with an emphasis on patient-centered and trauma-informed care. Additionally, the protocol discusses physician responsibility in documenting encounters and legal reporting, which may vary depending on location. We stress the importance of meeting the needs of the patient while prioritizing the safety of all involved. Additionally, the protocol provides a list of resources for the patient beyond medical care such as emergency housing, legal assistance, and food pantries. The overall purpose of this protocol is to provide coordinated response so that all providers may be consistent in caring for this vulnerable population.

## INTRODUCTION

*It is 1am in a single coverage emergency department (ED) when a 17-year-old female presents with her boyfriend complaining of stomach pain. Her boyfriend states the patient “just needs a pregnancy test” and wants to know how long this will take. You note that the patient is withdrawn with a flat affect and looks to her boyfriend for approval before answering questions. You observe that the boyfriend has the patient’s identification and states the bill can be sent to his address. He provides his email address and cell phone as her contact information. He interjects repeatedly to ask about the pregnancy test, stating they are in a hurry.*

You ask to examine the patient alone and the boyfriend and patient reluctantly agree. You complete a focused exam, including a pelvic examination, while the patient continues a videotelephone conversation with her boyfriend. You ask the patient to end the phone conversation, so you can speak with her privately. She reports being sexually active with multiple partners. She denies drug use. She is not from the area and has difficulty explaining how she supports herself. It is unclear whether she has any support from her family or friends. You are concerned that she is being exploited but you are unsure how to address your concern about human trafficking. You diagnose the patient with cervicitis and treat her in the ED. The pregnancy test is negative. The boyfriend returns and appears relieved at the news, quickly shuttling the patient out the door. Although the healthcare team recognized the controlling relationship between the patient and her boyfriend and the unstable social situation, the team did not know how to address its concern for possible human trafficking or what to do if she was verified as being a human trafficking victim.

Case background: This patient was actually 14 years old and had used a fake identification card. The “boyfriend” was actually 27 years old, and as her pimp he was anxious for her to get her back to work in sex trafficking. The physical exam occurred while the patient was clothed, and the physician did not see the cigarette burns along her bra line or the branding on her abdomen. She was a runaway and had been missing for over six months. Although a report was made to the child protective services agency after the ED encounter, the patient was not found.

The physician and nurses caring for the patient considered the possibility of human trafficking, but they did not know how to help the patient, how to broach the topic, or what resources were available to help her. The purpose of this article is to provide guidelines on the implementation of a human trafficking recognition and response program in the community hospital setting. The goals of the human trafficking program are to expertly assess the victims’ safety as they are being cared for as patients, to provide both medical care and social resources for human trafficking victims, and to advocate for their rights.

According to United States (US) federal law, human trafficking includes 1) sex trafficking: the recruitment, harboring, transportation, provision, obtaining, soliciting, or patronizing of a person for the purpose of a commercial sex act using force, fraud, or coercion, OR involving a child younger than 18 years; or 2) labor trafficking: the recruitment, harboring, transportation, provision, or obtaining of a person for labor or services, through the use of force, fraud, or coercion for the purpose of subjection to involuntary servitude, peonage, debt bondage, or slavery.^1^,^2^ Human trafficking includes a variety of offenses that range from domestic servitude to childhood survival sex, ie, children engaging in sexual activity in exchange for basic needs such as food and shelter. Approximately 20 million persons worldwide are victims of forced exploitation generating billions of dollars annually.^3^–^5^ Research estimates that between 100,000–300,000 children under 18 years old are at risk for commercial sexual exploitation annually, with the average age of entry into sex work in the US of 12–14 and 11–13 years of age for girls and boys, respectively. 6,7

Studies show that up to 88% of trafficked persons see a healthcare provider during their time in captivity.^8^–^10^ Lederer and Wetzel surveyed over 100 survivors of domestic sex trafficking and found that 63% of survivors reported going to an ED while being trafficked.^9^–^11^ Emergency physicians play a crucial role in serving the medical, psychiatric, and social needs of human trafficking survivors, as we are often their only access to medical care and resources. It is imperative to improve the recognition and response of the medical community to this vulnerable and underserved patient population.

Macias highlights the importance of trauma-informed, patient-centered care and how established policies and protocols can help physicians safely and appropriately respond to the complex healthcare needs of these patients.^4^ A trauma-informed approach recognizes the impact of trauma throughout the life of a patient, recognizes signs and symptoms of trauma, and responds appropriately while focusing on minimizing retraumatization.^4^,^12^ This approach is paramount for victims as it can lead to more positive interactions between healthcare providers and patients when providers have a better understanding of the effects of trauma on behavior and can help foster healing and recovery.^4^

The HEAL Trafficking and Hope for Justice Protocol Toolkit is a 44-page document developed in 2016 by experts in the field of human trafficking to guide providers in developing a human trafficking protocol.^13^ The healtrafficking.org website provides comprehensive protocol resources, as well as examples of tools and documents such as the Vera Institute of Justice “Out of the Shadows” tool, the Children’s Healthcare of Atlanta Institute on Healthcare and Human Trafficking guidelines, and the Dignity Health *Shared Learnings Manual*.^14^–^16^ These extensive protocols all require integration with community and systems-wide stakeholders, which may not be initially possible in all facilities. Here, we share our experience in narrowing the HEAL Trafficking Protocol Toolkit to develop a feasible, initial human trafficking recognition-and-response protocol for a location that does not yet have significant resources and community or system buy-in.

This paper reviews some of the current human trafficking literature and describes the implementation of the HEAL Toolkit based on our experience at an academic, urban, county ED serving 85,000 patients per year with a dedicated children’s ED serving 35,000 patients per year. At the time that we developed our protocol, faculty in our department did not have a set of tools or resources available to help recognize and assist this population, and there were no faculty members actively engaged in anti-human trafficking work as their primary niche. Our providers needed a guideline on how to address the needs of survivors.

### Human Trafficking in North Carolina

In 2017 the National Human Trafficking Hotline ranked North Carolina as eighth in the country for number of calls to the hotline with a total of 854 calls and 221 confirmed cases of human trafficking; these numbers have been steadily increasing over the past five years.^17^ Several factors contribute to the problem of human trafficking in North Carolina. First, its largest city Charlotte is home to several sports teams and hosts major sporting events. There has been a documented increase in human trafficking in US cities that host sporting events.^18^ Secondly, Charlotte is a refugee resettlement city and a transportation hub, meaning there is a vulnerable population and easy access to transport victims in and out of the area. Additionally, there are major businesses and tourism that result in a demand for sex work, including a large military presence with associated businesses that fuel sex trafficking and an agricultural community where vulnerable laborers can be easily hidden in rural areas while being exploited. There are an estimated 2200 homeless teenagers in the city of Charlotte; 33% are expected to have become victims of sexual exploitation within 48 hours of becoming homeless.^19^

## STEPS FOR PROTOCOL DEVELOPMENT-HOW TO GET STARTED

The HEAL Trafficking and Hope for Justice Protocol Toolkit helps healthcare providers develop a consistent response.^13^ The toolkit focuses on interacting with patients in a trauma-informed manner, which recognizes the impact of trauma on its victims and aims to avoid re-traumatization during interactions with these patients. The following paragraphs outline steps of protocol development.

### Step One: Understand Human Trafficking and Health Generally and Locally

Ideally all stakeholders are knowledgeable about health and trafficking and the scope of this problem locally. The HEAL Trafficking Toolkit lists several resources for education and networking.^13^ The National Human Trafficking Hotline can locate local service agencies where one can engage with individuals who regularly encounter victims of human trafficking.^17^ Attending trainings such as those sponsored by law enforcement agencies and networking with people or groups involved in anti-trafficking work can help one to characterize the local trafficking patterns. Additionally, legal contacts can help clarify anti-trafficking and mandatory reporting laws in each state.

We invited the ED faculty, learners, and ancillary staff to attend a lecture led by a law enforcement agent specializing in human trafficking. The session focused on explaining the types of trafficking, understanding the psychology of victims, and identifying and interviewing suspected victims. The agent discussed how to communicate with potential victims by using and understanding the language common within this lifestyle. For example, women involved in sex trafficking often refer to their trafficker as “boyfriend” or “daddy” and may refer to other women under the same man as “wifey.” The patient may refer to “turning a trick,” which is a term used to describe a sexual act for which payment is received.

### Step Two: Understand How Survivors Gain Assistance from Non-Medical Stakeholders in the Community

The HEAL Toolkit recommends creating a database of local multidisciplinary responders and lists several resources.^13^ Victims may need assistance with a variety of non-medical issues such as housing, counseling, legal services, and more. The National Human Trafficking Hotline (888-373-7888 or text “HELP” to 233733) can help in locating community-based, non-medical stakeholders.^17^ Our team located local anti-trafficking agencies, legal service providers and translation services, as well as housing and substance abuse resources. We collaborated with the leader of the domestic violence advocacy program in our hospital, a social worker, as well as a local law enforcement victim advocate. These contacts proved invaluable for our protocol development.

### Step Three: Organize the Medical Community to Provide a Safety Net for Survivors

The medical needs of human trafficking survivors will extend beyond the scope of the ED and include substance abuse and other mental health disorders, infections, reproductive health issues, injuries, and more.^3^,^4^,^21^,^22^ Individuals who specialize in these areas can add to the protocol, and also help create a multidisciplinary referral program. In the interest of a timely protocol roll-out, our team did not initially create an internal multidisciplinary treatment team. However, approximately six months later we did develop a team that includes a listserv of 45 individuals from multiple specialties.

### Step Four: Create and Convene an Interdisciplinary Protocol Committee

The authors of the HEAL Trafficking Toolkit recommend creating a committee comprised of both medical and non-medical stakeholders who will meet regularly to plan, implement, and revise the protocol.^13^ This should be an ongoing process as knowledge and service gaps are revealed and resource availability fluctuates. The evidence behind hospital protocols is limited, as this is an area that requires more research. This consensus-based recommendation has been implemented by individuals and organizations such as Dignity Health that are highly involved in anti-trafficking work.^16^ At the inception of planning and creating our protocol, only a few other specialties were involved. We learned that making this a truly collaborative approach from the start would have likely yielded more interest and participation in the project.

## PROTOCOL COMPONENTS

After following the steps for protocol development as outlined by the HEAL Trafficking Toolkit and gathering all the necessary resources, it is important that the following components are incorporated into the protocol.^13^

### Identifying Patients at Risk for Trafficking

There are currently no validated screening tools for use in the ED. Greenbaum, Shandro and several other experts in the field have published a variety of screening questions to identify persons involved in human trafficking. We adapted our screening questions from this published data, focused on both labor and sex trafficking.^3^,^8^,^21^,^22^ Victims and survivors are unlikely to self-identify. Therefore, one must commit to a criterion such as a positive answer to one of the screening questions, or any observed risk factors, that will trigger the healthcare team to do an in-depth screening.^3^,^20^,^21^

Each facility must determine how to identify their at-risk patients. Options for screening include observational screening, direct screening, or engaging those who self-identify. Our protocol lists “red flag” signs and symptoms recognizable through passive observation by anyone in contact with the patient, as well as specific indicators to look for during the medical assessment. These factors are highlighted in the Table, adapted from the Polaris Project.^17^ The Polaris Project is an anti-trafficking organization whose goal is to help trafficking victims and survivors, and pursue and prosecute traffickers.

Although our initial plan was to screen all patients during the triage process, we were unable to get the necessary agreement from nursing to implement this protocol. Currently we implement a focused screening based on observed risk factors. Patients who demonstrate red flags from the Table are questioned further to determine whether they are at risk for trafficking. If so, the treating physician activates the protocol.

### Interviewing High-Risk Patients

Providers should realize that victims of trauma, such as human trafficking survivors, may have emotional, physical, or cognitive reactions such as dissociation or depersonalization. These reactions may impede one’s ability to communicate effectively regarding the victim’s trauma.^23^ The goal is to establish trust and prevent re-traumatization. Avoid multiple interviewers or focusing on traumatic details that will not affect immediate care. Despite good intentions, excessive questioning by a physician, often in the setting of invasive exams or procedures, can exacerbate traumatic stress. Research has demonstrated that retraumatization may decrease the likelihood of patients achieving good health, adopting healthy behaviors, and returning for help.^12^

Interviewing the patient alone is ideal.^3^,^13^,^21^ Traffickers often accompany their victims, portraying themselves as friends or family, prohibiting the patient from asking for help.^3^,^4^,^13^,^22^ For minors, one can involve law enforcement and separate the victim from the trafficker if other methods of separation fail. For adults, providers can work with patients to determine whether forceful separation is a safe option, as a threatened trafficker may hinder the victim’s ability to return for help.^13^ It is also important to note that for patients who speak a foreign language, the provider should only use official interpreters to communicate with the patient.^3^,^13^,^21^

Currently at our institution, the provider interviews the patient. Suspected victims are interviewed alone, by as few individuals as possible. We encourage providers to mirror the language the victim uses in his or her self-identification, to maintain a non-judgmental tone, and to minimize questioning the patient about specific details of encounters that will not affect medical management. Our protocol prohibits use of companions as translators if one is suspicious of human trafficking, as it is possible that the companion is involved in their exploitation.

### Safety Considerations

Safety is a major consideration when working with victims of human trafficking, as traffickers want to avoid prosecution and may threaten or harm victims who are attempting to escape. The HEAL Trafficking protocol recommends involving hospital security in human trafficking trainings given the criminal element involved in trafficking.^13^

We focused mainly on how to address the immediate safety and security of our patients and staff. When a known or suspected victim of human trafficking presents, our registration team is instructed to have the patient listed under an alias, and staff is made aware that this patient is potentially in danger. Our physicians are encouraged to call upon our security officers if there is a direct threat. Additionally, we have the option of placing our ED on lockdown if a patient is in imminent danger and notifying the local police. We recommend patients turn off cell phones to limit contact with his or her controller and prevent location tracking. Despite our recommendations, providers need to collaborate with the patient to ensure that we are not jeopardizing safety with our efforts to intervene.

### Procedures for External Reporting

The HEAL Trafficking Protocol focuses on creating procedures for reporting that honor mandatory reporting laws, the Health Insurance Portability and Accountability Act (HIPAA), and autonomy for adult patients.^13^ In several states, trafficked persons can be prosecuted for crimes committed during their captivity. Involving law enforcement against the will of an adult patient when not legally mandated may violate HIPAA and result in unintended legal consequences, as well as a breach of trust between patient and physician.^21^ In North Carolina, healthcare providers are mandatory reporters for minors under the age of 18 suspected of being victims of human trafficking and this is reflected in our protocol. We encourage providers to involve the patient in the process and advise him or her of the agencies that will be notified. Anyone involved in a minor’s care who has suspicions of trafficking or abuse can make a report.

### Strategies for Responding to Patients Who Decline Assistance

Protocols should have resources for high-risk minors who do not meet any criteria for legal involvement, or adults who decline assistance. Patients should not be pressured; instead, they should be offered resources, such as the National Human Trafficking Hotline Number.^17^ Information should be given in a discreet manner that can be hidden if necessary.^21^ We compiled a list of external resources to address potential issues including legal and immigration assistance, housing, food insecurity, and substance abuse. We encourage our team to ask survivors what assistance they want, as they may decline help initially, return to their situations repeatedly, and only later be ready to exit permanently.^13^

### Procedures Regarding Documentation

Documentation in the medical record can have legal ramifications, and guidelines should be created in consultation with legal experts.^13^ Most trafficked persons experience some degree of violence while being trafficked.^5^ Given the complex nature of trauma, survivors often suppress memories or withhold information.^23^ These factors can lead to accounts of events changing over time, which may negatively impact legal credibility. We were advised not to alter our patient interview or documentation with potential legal consequences in mind and would encourage the reader to consult with their institution’s legal team.

### Guidelines for Forensic Examination

Protocols should outline the details of the interviewing, examination, and documentation process for the forensic examination.^13^ Our facility has sexual assault nurse examiners (SANE) on call, who are responsible for the forensic examination and evidence collection. Any patient who has experienced sexual assault within 72 hours of presentation should be offered a SANE exam. Physicians are encouraged to participate in the examination and questioning by the SANE nurse to minimize retraumatization.

## CONCLUSION

### Sustainable Change: How to Maintain Momentum and Improve the Consistency of Care

It is crucial that emergency physicians be educated on how to identify victims and how to address their unique set of medical and social needs. We felt that having a protocol in place would be best to ensure that survivors and at-risk patients are treated appropriately and in a standardized manner regardless of the experience of the provider.

We are currently expanding our task force and our response to add medical treatment protocols and measures to make our response trauma-informed and unique to this patient population. Ideally, we will ultimately have a protocol and an extensive treatment and referral plan to meet the needs of all survivors.

## Figures and Tables

**Table:** Red Flag Indicators of Potential Human Trafficking.^17^

**Poor Mental Health or Abnormal Behavior:** The individual(s) in question: Is fearful, anxious, depressed, submissive, tense, or nervous/paranoid Exhibits unusually fearful or anxious behavior after bringing up law enforcement Avoids eye contact
**Poor Physical Health:** The individual(s) in question: Lacks health care Appears malnourished Shows signs of physical and/or sexual abuse, physical restraint, confinement, or torture
**Lack of Control:** The individual(s) in question: Has few or no personal possessions Is not in control of his/her own money, no financial records, or bank account Is not in control of his/her own identification documents (ID or passport) Is not allowed or able to speak for themselves (a third party may insist on being present and/or translating)
**Common Work and Living Conditions:** The individual(s) in question: Is not free to leave or come and go as he/she wishes Is under 18 and is providing commercial sex acts Is in the commercial sex industry and has a pimp/manager Is unpaid, paid very little, or paid only through tips Works excessively long and/or unusual hours Is not allowed breaks or suffers under unusual restrictions at work Owes a large debt and is unable to pay it off Was recruited through false promises concerning the nature and conditions of his/her work High security measures exist in the work and/or living locations (e.g., opaque windows, boarded up windows, bars on windows, barbed wire, security cameras, etc.)
**Other:** The individual(s) in question: Claims of just visiting and inability to clarify where he/she is staying/address Lack of knowledge of whereabouts and/or do not know what city he/she is in Loss of sense of time Has numerous inconsistencies in his/her story
